# Supplementary figures and images for: Measuring leadership an assessment of the Multifactor Leadership Questionnaire
Source: PLoS One. 2021 Jul 22;16(7):e0254329. doi: 10.1371/journal.pone.0254329 (PMC8297756; doi:10.1371/journal.pone.0254329)

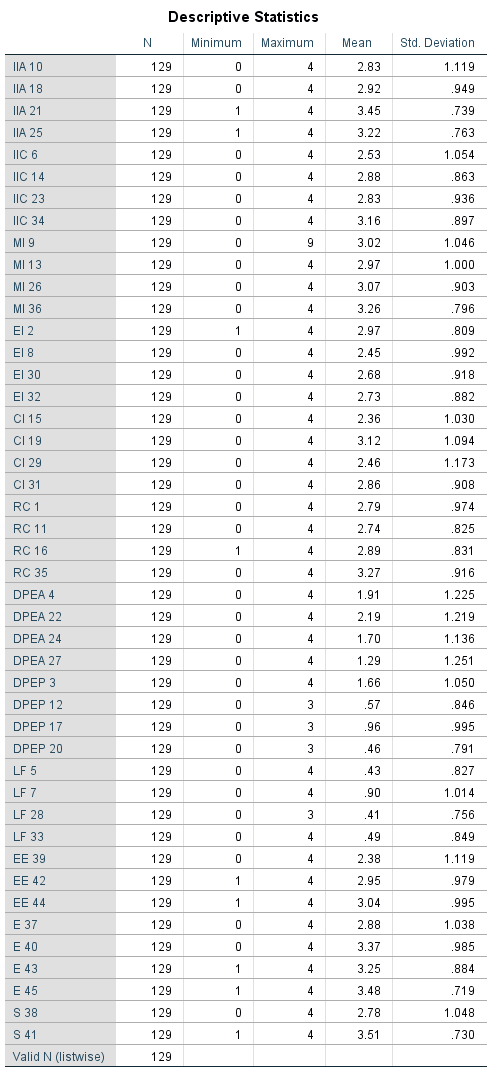

Supplement: S1 File — (DOCX) [file pone.0254329.s002.docx]

**Correlations matrix**


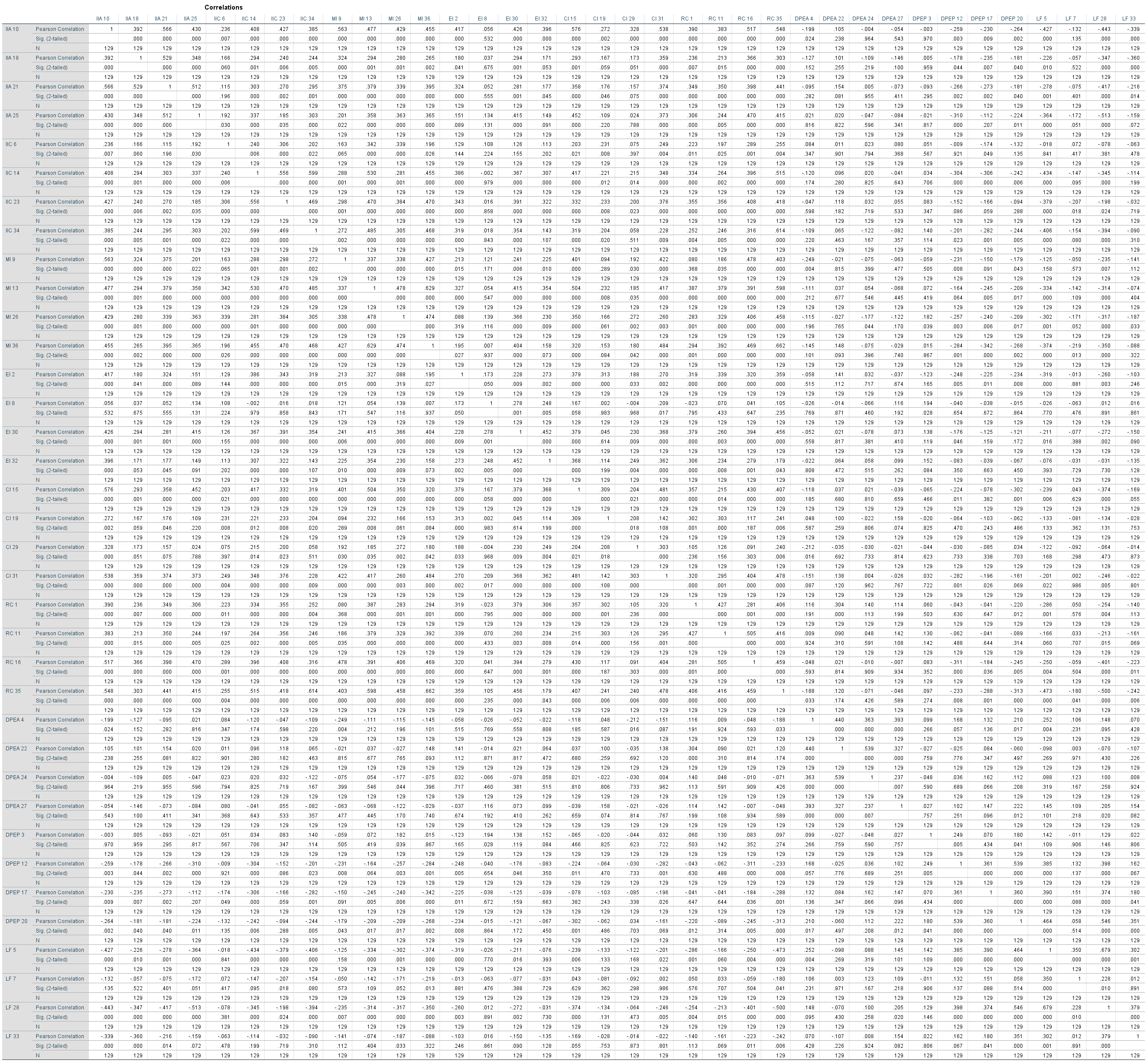

Supplement: S2 File — (DOCX) [file pone.0254329.s003.docx]
